# An introduction to niche construction theory

**DOI:** 10.1007/s10682-016-9821-z

**Published:** 2016-02-03

**Authors:** Kevin Laland, Blake Matthews, Marcus W. Feldman

**Affiliations:** School of Biology, Sir Harold Mitchell Building, University of St Andrews, St Andrews, Fife, KY16 9TF UK; Aquatic Ecology Department, Center for Ecology, Evolution and Biogeochemistry, Eawag, Seestrasse 79, 6047 Kastanienbaum, Switzerland; Department of Biology, 385 Serra Mall, Stanford University, Stanford, CA 94305 USA

**Keywords:** Niche construction, Niche construction theory, Ecological inheritance, Ecosystem engineering, Cultural niche construction

## Abstract

Niche construction refers to the modification of selective environments by organisms. Theoretical and empirical studies of niche construction are increasing in importance as foci in evolutionary ecology. This special edition presents theoretical and empirical research that illustrates the significance of niche construction to the field. Here we set the scene for the following papers by (1) discussing the history of niche construction research, (2) providing clear definitions that distinguish niche construction from related concepts such as ecosystem engineering and the extended phenotype, (3) providing a brief summary of the findings of niche construction research, (4) discussing the contribution of niche construction and ecological inheritance to (a) expanded notions of inheritance, and (b) the extended evolutionary synthesis, and (5) briefly touching on some of the issues that underlie the controversies over niche construction.

## Introduction

Niche construction is the process whereby organisms actively modify their own and each other’s evolutionary niches (Odling-Smee et al. 2003). Examples include the building of nests, burrows, mounds, and other artifacts by animals; the alteration of physical and chemical conditions; the creation of shade, influencing wind speed; and the alteration of nutrient cycling by plants. When such modifications alter natural selection pressures, evolution by niche construction is a possible outcome.

The niche construction perspective was brought to prominence through the writings of Harvard biologist Richard Lewontin, although related ideas can be found in earlier work by Schrodinger (1944) and Waddington (1969). Lewontin (1983) pointed out that organisms do not passively adapt to conditions in their environment, but actively construct and modify environmental conditions that may influence other environmental sources of selection. Lewontin’s articles were the first to address what we now call ‘niche construction’, but were largely interpreted as a component of Lewontin’s critique of adaptationism.

Oxford biologist John Odling-Smee (1988) was the first to coin the term ‘niche construction’, the first to make the argument that niche construction should be recognized as an evolutionary process, and the first to introduce the concept of ‘ecological inheritance’. These ideas gained prominence among evolutionary biologists and ecologists with Odling-Smee et al.’s (1996) article in *The American Naturalist*. Models investigating the ecological and evolutionary ramifications of niche construction followed (Laland et al. 1996, 1999; Kerr et al. 1999). Further developments came with the publication of Odling-Smee et al’s. (2003) monograph *Niche Construction: the Neglected Process in Evolution*. This book proposed a more dynamic niche concept, namely an ‘evolutionary niche’, which is ‘the sum of all the natural selection pressures to which the population is exposed’. It also demonstrated how organisms can construct developmental environments for their offspring, or modify environmental states that will be experienced by other descendants, a process known as ‘ecological inheritance’ (Odling-Smee 1988; Odling-Smee et al. 2003, 2013). In recent years ecological inheritance has been widely recognized as a core component of extra-genetic inheritance, and has become central to attempts within evolutionary biology to broaden the concept of heredity beyond transmission genetics (Danchin et al. 2011; Bonduriansky 2012; Badyaev and Uller 2009). The development of many organisms, and the recurrence of traits across generations, depends critically on the construction of developmental environments by ancestral organisms (Badyaev and Uller 2009; Sultan 2015).

The concepts of evolutionary niches and ecological inheritance are central to understanding four key tenets of niche construction theory: (1) organisms modify environmental states in nonrandom ways, thereby imposing a systematic bias on the selection they generate, and allowing organisms to exert some influence over their own evolution (Odling-Smee et al. 2003; Laland 2014); (2) ecological inheritance strongly affects evolutionary dynamics (Odling-Smee et al. 2013), and contributes to parent-offspring similarity (Danchin et al. 2011; Bonduriansky 2012; Badyaev and Uller 2009); (3) acquired characters and byproducts become evolutionarily significant by affecting selective environments in systematic ways, and (4) the complementarity of organisms and their environments (traditionally described as ‘adaptation’) can be achieved through evolution by niche construction (Odling-Smee et al. 2003). These findings suggest that evolution by niche construction may be an under-appreciated process in nature, which warrants additional experimental and comparative tests on natural populations (Matthews et al. 2014).

## Definitions of niche construction

The definition of niche construction is purposely broad, encompassing how selection is affected both by physical changes that organisms bring about in their environments (‘perturbational niche construction’) and by when they move in space and are exposed to new conditions (‘relocational niche construction’) (Odling-Smee et al. 2003). Part of the reason for this broad definition was that seemingly trivial environmental modifications, relative to externally driven environmental forcing or environmental heterogeneity, might nevertheless produce important selection pressures. Examples are the soil-generating consequences of snails that consume endolithic lichens and thereby support a desert ecosystem (Jones et al. 1997), and the seabird guano that transforms island shrub to lush grassland (Croll et al. 2005). Neither of these niche-constructing effects are adaptations (or extended phenotypes), yet both have major ecological, and plausibly evolutionary, consequences. It is far more obvious that the beaver’s dam is of evolutionary and ecological importance than that the beaver’s dung is, although the latter is actually a real possibility. A pressing challenge is to quantify, and determine the relative importance of, niche construction in natural populations.

Matthews et al. (2014) propose a set of criteria (simplified here) to test for the presence of niche construction (Criteria 1 and 2) and to determine when it affects evolution (Criterion 3):

1. An organism must significantly modify environmental conditions;
2. Organism-mediated environmental modifications must influence selection pressures on a recipient organism;
3. There must be an evolutionary response in at least one recipient population caused by the environmental modification.

Criteria 1 and 2 are sufficient for the definition of niche construction (Odling-Smee et al. 2013), while the third criterion is a test of evolution by niche construction. Implementation of criterion 1 depends on how the term ‘significantly’ is interpreted. Strictly, everything that an organism does changes the environment to some degree. Whether a form of environmental modification is recognized as ‘niche construction’ requires a pragmatic judgment by the researcher as to whether the modification is relevant to their research. Evolutionary ecologists will typically consider criteria 1 and 2 together, and hence the key issue is whether the environmental modification is sufficiently substantial in scale, duration and impact to plausibly affect selection.

Niche construction has a superficial similarity to Richard Dawkins’ (1982) concept of the ‘extended phenotype’, although in practice the latter is a narrower concept. For Dawkins, extended phenotypes are adaptations expressed outside of the body of the individual whose genes underlie a constructing trait. Clearly, there is overlap between these concepts; for instance, animal artifacts are both examples of niche construction and extended phenotypes. However, there are at least three important differences.

First, in the case of an extended phenotype, the focus is restricted to the selective feedback from environmental modification to the gene or genes responsible for the environmental modification. For instance, alleles underlying beaver dam building are differentially favoured according to the fitness consequences of their impact on dam building. Niche-construction theory, in contrast, draws attention to the fact that there is also selective feedback to other loci, potentially unrelated to the genes underlying the modifications, The dam, for instance, is the source of modified selection on a host of beaver traits, ranging from social behavior to vulnerability to disease (Naiman et al. 1988).

Second, this modified selection remains so long as the dam, lake and lodge persist, which can be often be longer than the lifetimes of the beavers that manufactured them (Naiman et al. 1988). Unlike extended phenotype theory, niche construction theory recognizes this legacy of modified selection as both an additional component of inheritance (‘ecological inheritance’) and an influence on evolutionary dynamics (Odling-Smee et al. 1996; Laland et al. 1999).

Third, in the case of an extended phenotype, the phenotype that underlies the organism-mediated modification of the environment must have a genetic basis, and be the target of the altered selection regime caused by the environmental modifications (Dawkins 2004; Brodie 2005). While not disputing the importance of extended phenotypes, niche construction theory (Odling-Smee et al. 2003) neither requires that the traits underlying specific environmental modifications have a strong genetic basis (e.g. they can be acquired characters) nor that the same traits develop strong associations with fitness. Acquired characters are typically not extended phenotypes, yet they can generate selective feedback if they are expressed in niche construction. For example, through the farming and consumption of starchy foods, human cultural practices have generated selection for multiple copies of the salivary amylase gene (AMY1) in many human populations (Perry et al. 2007). Byproducts can also become evolutionarily significant by affecting selective environments in systematic ways, as in the Negev desert snails example (Jones et al. 1997). Odling-Smee et al. (2003, 2013) suggest that covariance between fitness and phenotype can frequently build up between species due to organism-mediated modifications to both biotic and abiotic environmental conditions (Matthews et al. 2014).

Typically, evolutionary biologists assume that if a niche-constructing activity generates evolutionary feedback to the constructor, then it must be an adaptation, but this need not be the case. Theoretical work shows that byproducts can be consequential for the constructor’s own evolution where, through niche construction, they precipitate bouts of selection in the constructor population by inducing selection on other traits, and hitchhiking to fixation on the back of this selection that they generate (Silver and Di Paolo 2006; Rendell et al. 2011). Here there is *selection of* the niche-constructing trait, but not *selection for* it (Williams 1966; Sober 1984).

Odling-Smee et al. (2003) present numerous examples of niche construction, for instance, the nests and burrows of animals, but in many such cases it has yet to be demonstrated that the constructing activity has fed back to influence the evolution of the constructor, or any other species. There are, however, specific well-studied examples of niche construction that clearly demonstrate this evolutionary feedback. For instance, Callahan et al. (2014) show experimentally that niche construction can evolve rapidly, under a broad range of conditions, in microbial populations. Likewise Buser et al. (2014) demonstrate how yeast (*Saccharomyces cerevisiae)* can modify its fruit environment to attract Drosophila, thus facilitating its own propagation. Other examples include shell modification by hermit crabs (Laidre 2012), and evolution of flammability (Schwilk 2003) and germination time (Donohue 2013) in plants.

## The niche-construction perspective

The niche-construction perspective entails that niche construction be regarded as a fundamental evolutionary process in its own right:

The organism influences its own evolution, by being both the object of natural selection and the creator of the conditions of that selection (Levins and Lewontin 1985, p 106).

Evolution thus entails networks of causation and feedback in which previously selected organisms drive environmental changes, and organism-modified environments subsequently select for changes in organisms (Odling-Smee et al. 2003; Laland et al. 2011). In recognizing developmental processes as evolutionary causes, this perspective is intellectually aligned with other developments within evolutionary biology, including ‘developmental systems theory’ (Oyama et al. 2001), the active role of behavior (Bateson 1988), developmental plasticity (West-Eberhard 2003; Kirschner & Gerhart 2005), ‘evo-devo’ (Arthur 2004; Müller 2007) and ‘eco-devo’ (Gilbert and Epel 2009; Sultan 2015), and is also central to the argument for an extended evolutionary synthesis (Laland et al. 2014, 2015). It is also aligned with eco-evolutionary dynamics theory because feedbacks between environmental modification and selection pressures may develop when the timescales of ecological and evolutionary dynamics overlap (Post and Palkovacs 2009; Matthews et al. 2014).

While evolving in a new environment, organisms can change their phenotype (i.e. adaptive phenotypic plasticity) and/or modify their environment (e.g. niche construction). As both of these capabilities incur costs and involve adaptive specializations, tradeoffs between plasticity and niche construction may arise within organisms. At the same time, plasticity is a major source of niche construction, and hence species might be expected to vary in the magnitude of their niche construction, and the extent to which it is underpinned by plasticity. Hence the tradeoff between plasticity and niche construction may be manifest within, but not between, species. An important challenge for the future will be to devise means of quantifying niche construction.

Niche construction is not simply a source of environmental change but is a driver of selection that may produce novel evolutionary outcomes. For instance, in building a nest, an organism creates selection for the nest to be defended, maintained, and regulated, as well as for other organisms to inhabit it, or dump eggs in it. Such adaptive responses may generate parallel evolution in independent lineages, long-term trends, patterns of diversification, and variation in evolvability (Laland et al. 2015). These predictions can be investigated, at micro-evolutionary or macro-evolutionary scales, using experimental and comparative phylogenetic methods. Evolution by niche construction may lead to more rapid adaptation than evolution by selection from non-modifiable environmental conditions, a hypothesis that could be testable in natural or in silico experiments (Zaman et al. 2014). These examples illustrate how the niche-construction perspective can generate novel predictions, and open up new lines of enquiry.

While the occurrence of niche construction is not disputed (its recognition goes back to Darwin’s classic books on earthworms and corals) the niche-construction perspective nonetheless remains controversial. Researchers differ over to what extent niche construction requires changes in evolutionary theory (e.g. compare Laland et al. 2014 to Wray et al. 2014). In particular, the claim that niche construction is an evolutionary process, and a source of adaptation, has ignited controversy. These issues are discussed by Laland and Sterelny (2006), on the website http://lalandlab.st-andrews.ac.uk/niche/ and by Scott-Phillips et al. (2014). The latter is a collaboration between critics of the niche-construction perspective and one of its advocates. The authors’ disagreements reflect a wider dispute within evolutionary theory over whether the neo-Darwinian synthesis is in need of reformulation, as well as different usages of some key terms (e.g., evolutionary process).

## Niche construction in evolutionary biology and ecology

An extensive body of formal theory explores the evolutionary consequences of niche construction and its ramifications for evolutionary biology and ecology. For instance, under niche construction, genes or phenotypes can fix that would, under standard evolutionary theory, be deleterious (Laland et al. 1996, 1999; Creanza and Feldman 2014). Niche construction can also create or eliminate equilibria, affect evolutionary rates, cause evolutionary time lags, generate momentum, inertia, autocatalytic effects, catastrophic responses to selection, and cyclical dynamics (Laland et al. 1996, 1999; Creanza and Feldman 2014). Niche-constructing traits can drive themselves to fixation by creating statistical associations with recipient traits (Silver and Di Paolo 2006), and facilitate the evolution of cooperation (Lehmann 2008; van Dyken and Wade 2012). It can also regulate environmental states, allowing some organisms to persist in otherwise inhospitable conditions, thus facilitating range expansion and affecting carrying capacities (Kylafis and Loreau 2008; Krakauer et al. 2009). Finally, theory suggests that niche construction can drive coevolutionary events, exacerbate and ameliorate competition, affect the likelihood of coexistence, and produce macroevolutionary trends (e.g. Krakauer et al. 2009).

While niche construction theory has focused on how organisms modify selection, evolution by niche construction may occur when environmental modifications affect the interaction between selection, random drift, and gene flow. By moving in space, organisms may expose themselves to novel selection (relocatory niche construction), but these movements may influence gene flow among demes in a metapopulation. Likewise, through manufacturing habitat, perturbatory niche construction can affect carrying capacities (Gurney and Lawton 1996), which affects drift by changing effective population sizes. In this way, organisms might influence the dynamics and outcome of drift, and the balance between drift and selection.

In recent years, efforts have been made to operationalise the niche construction concept within ecology. Odling-Smee et al. (2013) describe some of the ecological and evolutionary impacts on ecosystems of niche construction, and illustrate how it triggers ecological and evolutionary feedbacks, leaving detectable ecological signatures that can be investigated. Matthews et al. (2014) propose an operational framework to evaluate comparative and experimental evidence for the evolutionary consequences of niche construction, and suggest research that can improve understanding of ecological and evolutionary dynamics. For example, in a recent experiment, Matthews et al. (2016) found feedbacks between the ecosystem modifying activities of adult fish, and the fitness relationships among juveniles in a subsequent generation.

Niche construction theory can provide new perspectives on classic ecological and evolutionary questions including some pressing practical matters (Boogert et al. 2006). For example, Kylafis and Loreau (2011) introduce a model of two consumers that compete for one limiting resource, and one predator. They show how niche construction modifies the traditional niche-deteriorating impacts of its agent or of competing species, and hence affects the potential for species coexistence. Niche construction may also be involved in generating local spatial effects that can allow a population to augment its own range expansion (Kylafis and Loreau 2008), linking eco-evolutionary feedbacks to the process of adaptive radiation. Rather than lineages simply diversifying to “fill” available niches, niches themselves may be diversifying (Erwin 2005), a process subsequently termed “self-propagating adaptive radiation.” Classic models of ecological speciation (Doebeli and Dieckmann 2003) incorporate feedbacks between ecological and evolutionary dynamics in relatively simple ways compared to those feedbacks envisioned by niche-construction theory. In such models, efficiency of resource utilization evolves locally and increases the strength of frequency dependence in spatially localized ecological interactions, sometimes leading to disruptive selection (Doebeli and Dieckmann 2003). However, another possibility is that organisms locally modify their environments, and thereby influence the spatial and temporal structure of selection on traits that underlie these modifications, or on other traits that could influence evolutionary responses. Exploring a broader range of different possible feedback scenarios (Han and Hui 2014), beyond the classic arguments of frequency-dependent selection, might yield new insights into the origin and maintenance of diversity (see also Krakauer et al. 2009).

## Niche construction and the human sciences

Niche construction theory has had a particular impact in the human sciences, including biological anthropology (Anton et al. 2014), archaeology (O’Brien and Laland 2012), and psychology (Flynn et al. 2013). Niche construction is now recognized to have played important roles in human evolution (Kendal et al. 2011; Anton et al. 2014), including the evolution of cognitive capabilities (Bickerton 2009). It is clear that humans possess an extremely potent capability to control, regulate, construct and destroy their environments, and that this is generating some pressing current problems (e.g. climate change, deforestation, urbanization). Human scientists have been attracted to the niche construction perspective because it recognizes human activities as directing human evolution (Odling-Smee et al. 2003; O’Brien and Laland 2012).

Mathematical models reveal that niche construction due to human cultural processes can be as potent as niche construction that has evolved through biological evolution, and establish that cultural niche construction can modify selection on human genes and drive evolutionary events (Laland et al. 2001; Ihara and Feldman 2004; Creanza and Feldman 2014). There is now little doubt that human cultural niche construction has co-directed human evolution (Laland et al. 2010). Humans have modified selection, for instance, by dispersing into new environments with different climatic regimes, devising agricultural practices or domesticating livestock. The best-researched example is the finding that dairy farming created the selection that led to the spread of alleles for adult lactase persistence (Aoki 1986; Feldman and Cavalli-Sforza 1989; Gerbault et al. 2011). Analyses of the human genome have identified many genes subject to recent selection, selection that in many cases may be due to changes in the cultural niche. The lactose persistence example may be representative of a very general pattern of gene-culture coevolution over the last 20,000 years.

## Introduction to the articles in this special edition

One claim made by niche construction theory is that it provides additional conceptual tools with which to integrate evolution and ecology more effectively (Odling-Smee et al. 1996, 2003, 2013, Matthews et al. 2014). This is particularly relevant to the integration of evolutionary biology with ecosystem science, which historically has made little use of evolutionary principles and methodologies (O’Neill et al. 1986; Matthews et al. 2011).

Physiologist Scott Turner (this volume) identifies organismal agency as the key distinction between traditional evolutionary perspectives and niche construction theory. For Turner, agency in living systems is an expression of a fundamental property of life— homeostasis. In order to stay alive and maintain order in their bodies and immediate surrounds, organisms must act on their environment, and change it in a directional manner. The physiology of organisms, at various levels of organization from the cell to the colony, extends beyond its immediate boundaries—what Turner calls an “extended physiology” (Turner 2000). Organisms can thus be viewed as actively maintaining a nested series of adaptive boundaries to ensure suitable homeostatic relations. Turner emphasizes how the maintenance of extended homeostasis requires active agency on the part of the organism, and this must be informed—it is inherently purposive and demands some kind of ‘cognition’.

The bacterium *Pseudomonas fluorescens* is widely used as a model system for studying experimental evolution, including the evolution of niche construction (Callahan et al. 2014). Claire-Marie Loudon, Blake Matthews, Duygu Sevgi Sevilgen and Bas Ibelings (this volume) argue that it may also provide an opportunity to test predictions from both niche construction theory and dissipative systems theory. These authors examine how manipulating two aspects of niche construction by *Pseudomonas* affected the development of diversity of the bacterial community and its ability to dissipate the oxygen gradient (and thereby increase entropy). As predicted, system diversity increased because of evolution by niche construction, and along with it the rate of oxygen dissipation increased. This *Pseudomonas* system is also the inspiration for a model of the origins of developmental programs by Emma Wolinsky & Eric Libby (this volume), who show how genotypes can evolve sensitivities to the environmental conditions that they construct.

While theory suggests that niche construction can affect the accumulation and release of cryptic genetic variation (Laland et al. 1996, 1999; Odling-Smee et al. 2003; Laland and Sterelny 2006), few experimental studies have tested this hypothesis. Emilie Snell-Rood, Melissa Burger, Quinton Hutton, and Armin Moczek (this volume) suggest that parental niche construction can buffer offspring from environmental stress, for instance, by providing food or shelter, ameliorating the negative effects on fitness of new mutations, and resulting in the accumulation of cryptic genetic variation. They discuss this idea using the case of *Onthophagus* horned beetles, adult females of which construct a benign developmental niche for their offspring by constructing a brood ball of dung in which they oviposit a single egg, which provides the food that larvae need to complete larval development and metamorphosis. Snell-Rood and colleagues consider whether natural variation in parental behaviour is tied to the release of cryptic genetic variation when reared under scenarios that mimic the removal of parental investment. They illustrate how it would be possible to induce novel mutations experimentally and test the extent to which parental investment buffers selection on new mutations. Provisional data provide partial support for the behavioral buffering hypothesis, but also suggest that such buffering may vary in complex ways with specific mutations and traits.

Julia Saltz and her colleagues (this volume) note that while the terms ‘social niche’ and ‘social niche construction’ are starting to be deployed there is considerable variation in their usage. Saltz et al. bring clarity to these issues by proposing working definitions. They characterize the social niche as the set of social environments in which the focal individual has non-zero inclusive fitness. Only where a focal individual’s phenotype, as expressed in its social environment, results in the focal individual having non-zero inclusive fitness in that social environment, is the social environment part of the focal individual’s social niche. Social niche construction describes the situation in which individuals, singly or collectively, influence the composition and dynamics of their social environments, for instance, by choosing whom they associate with or by acting in such a way as to change the composition of their social environment. This focus raises a number of questions that historically have received little attention.

A longstanding challenge for evolutionary biology is to understand the mechanisms that support the evolution of cooperation. Brian Connelly, Katherine Dickinson, Sarah Hammarlund, and Benjamin Kerr (this volume) demonstrate that cooperation can persist by hitchhiking with beneficial non-social adaptations. Cooperators play an active role in this process. In spatially structured environments, clustered cooperator populations reach greater densities, creating mutational opportunities to gain beneficial non-social adaptations, and allowing cooperation to increase. However, once adaptive opportunities have been exhausted, the opportunity to spread ends, and cooperators are displaced by adapted defectors. Using an agent-based model, Connelly et al. demonstrate that the selective feedbacks, which are created as populations construct their local niches, can maintain cooperation indefinitely.

Nicole Creanza, Laurel Fogarty, and Marcus Feldman (this volume) propose that selection pressure on the size and complexity of birdsong repertoires may have facilitated the construction of a niche in which learning, sexual selection, and song-based homophily co-evolve. Creanza et al. analyze the relationship between the number of syllables in a species-typical repertoire and the length of the song-learning program, finding significantly larger repertoire sizes in open-ended compared to closed-ended learners. They go on to use a mathematical model to examine the interactions between the culturally transmitted trait of song repertoire size, the evolved capacity for song learning in adulthood, and mating preference for a song heard early in life. Underpinning the correlation between open-ended or closed-ended learning and repertoire size is a form of cultural niche construction in which a costly biological trait (open-ended learning) can spread in a population (or be lost) as a result of direct selection on an associated cultural trait (song repertoire size). Culturally transmitted song can be an important niche-constructing trait, influencing the spread of other traits that are likely to have genetic underpinnings, such as those that affect neural development and mating preferences.

This special edition ends with two articles on human niche construction. Archaeologist Bruce Smith (this volume) suggests that the human transition from hunting and gathering to food production economies provides an excellent opportunity to compare the relative strength of explanatory frameworks for domestication based on standard evolutionary theory with an alternative derived from niche construction theory. He shows how archaeological and paleo-environmental records from both eastern North America and northern South America contradict the assumption of traditional explanatory frameworks that environments change and species adapt. Conversely, explanations based on niche construction theory are well supported in both regions. Smith sees initial domestication as a lengthy evolutionary process that involves the complex interplay of a diverse array of environmental and cultural variables. He shows that initial domestication took place in settings where climatic fluctuation was limited enough, and biotic communities were rich and varied enough, to allow human societies to maintain sustainable settlements, which afforded the time and resources to experiment with domesticating plants and animals.

Melinda Zeder (this volume) proposes domestication as an excellent model system for testing predictions from niche construction theory. Like Smith (this volume), Zeder sees advantages to niche-construction theory over explanatory frameworks grounded in more traditional neo-Darwinian theory, for understanding initial domestication. These advantages stem from niche-construction theory’s focus on reciprocal causation, and the capacity of niche-constructing species to modify the niches of other species that share constructed environments. For Zeder, this provides a useful framework within which to understand the co-evolutionary mechanisms that lead to domestication, and the role of human intentionality. She concludes that domestication provides a valuable model system for niche-construction theory, and that data are consistent with predictions from the niche construction literature.

Collectively, the articles illustrate the diverse research programs that niche construction theory is starting to influence, and provide concrete evidence that the perspective is proving useful within evolutionary ecology.
